# Local Zoledronate Administration Modulates Periapical Lesion Development in Immunologically Distinct Rat Strains

**DOI:** 10.3390/dj14070393

**Published:** 2026-06-26

**Authors:** Tamara Milunovic, Milos Papic, Mirjana V. Papić, Miona Vuletic, Aleksandra Misic, Dejan Zdravkovic, Jovan Rakic, Ksenija Vucicevic, Marina Miletic Kovacevic, Milica Popovic, Slobodanka Mitrovic, Biljana Ljujic, Suzana Zivanovic

**Affiliations:** 1 Department of Dentistry, Faculty of Medical Sciences, University of Kragujevac, 34000 Kragujevac, Serbia; tamara.vucicevic@yahoo.com (T.M.); mira.radovic@hotmail.com (M.V.P.); miona91kg@gmail.com (M.V.); alek.vlaskovic@gmail.com (A.M.); zdravkovicdejan91@yahoo.com (D.Z.); jovanrakic88@yahoo.com (J.R.); milicapopovic75@gmail.com (M.P.); suzanazivanovic91@yahoo.com (S.Z.); 2 Department of Pharmacy, Faculty of Medical Sciences, University of Kragujevac, 34000 Kragujevac, Serbia; ksenija.vucicevic.kg@gmail.com; 3 Department of Histology and Embryology, Faculty of Medical Sciences, University of Kragujevac, 34000 Kragujevac, Serbia; marina84kv@gmail.com; 4 Department of Pathology, Faculty of Medical Sciences, University of Kragujevac, 34000 Kragujevac, Serbia; smitrovic@fmn.kg.ac.rs; 5 Department of Genetics, Faculty of Medical Sciences, University of Kragujevac, 34000 Kragujevac, Serbia; bljujic74@gmail.com

**Keywords:** periapical lesions, zoledronate, immune response, bone remodeling

## Abstract

**Background/Objectives**: The objective of this study was to investigate the effects of local zoledronate treatment during periapical lesion development on inflammatory and bone remodeling responses in two immunologically distinct inbred rat strains, Dark Agouti (DA) and Albino Oxford (AO). **Methods**: Periapical lesions were induced in the mandibular first molars of AO and DA rats (*n* = 44) by pulp exposure. Animals were assigned to four groups: DA + zoledronate, DA + saline, AO + zoledronate, and AO + saline. Zoledronate (0.15 mg/kg) or saline was locally administered on days 0, 7, 14, and 21 during lesion development. Animals were sacrificed on day 28. Mandibles were analyzed radiographically and histologically for lesion size, while osteogenic activity was assessed by osteocalcin immunohistochemistry. Gene expression was evaluated by qRT-PCR, and systemic oxidative stress parameters were analyzed spectrophotometrically. Statistical analysis included parametric or non-parametric tests according to data distribution, with significance set at *p* < 0.05. **Results**: Zoledronate -treated AO rats exhibited smaller periapical lesions and higher radiographic grayscale density than DA rats (*p* < 0.05). Histological analysis confirmed the radiographic findings and demonstrated smaller lesion areas in AO rats. Osteocalcin expression was significantly higher in AO rats (*p* < 0.05), indicating increased osteogenic activity. At the molecular level, DA rats showed higher expression of TNF-α and IL-1β, whereas AO rats exhibited higher expression of IL-10 and IL-4 (*p* < 0.05). In addition, expression of osteoclastogenic factor RANKL was significantly lower in AO rats than in DA rats (*p* < 0.05), while OPG expression showed a non-significant tendency toward higher levels in AO rats. Systemic redox analysis demonstrated lower NO_2_^−^ and O_2_^−^ levels in zoledronate-treated AO rats, whereas no significant differences were observed in the remaining oxidative stress parameters. **Conclusions**: Following local zoledronate treatment during lesion development, Th2-dominant AO rats exhibited reduced inflammatory responses and increased osteogenic activity compared with Th1-dominant DA rats. In contrast, DA rats primarily demonstrated attenuation of osteoclastogenic signaling without comparable osteogenic responses. These findings indicate that the biological effects of local zoledronate treatment in developing periapical lesions are influenced by the host immune phenotype.

## 1. Introduction

Periapical lesions represent chronic inflammatory diseases of microbial origin that develop as a result of complex host immune and inflammatory responses to infection of the root canal system. These lesions are characterized by localized bone resorption and destruction of periapical tissues, frequently resulting in chronic apical periodontitis and, if left untreated, progressive alveolar bone loss [1,2]. Management of periapical lesions aims not only to control infection but also to limit inflammatory bone destruction and support periapical tissue repair. Conventional endodontic therapy, involving thorough chemo-mechanical debridement and hermetic root canal obturation, is still the standard approach for managing these lesions. However, the healing outcome of periapical lesions is not solely dependent on effective microbial control but is also significantly influenced by the host’s immune and inflammatory responses [3].

Bisphosphonates are a class of drugs widely used to treat various bone-related disorders, including osteoporosis, Paget’s disease, and metastatic bone disease [4]. Their primary mechanism involves inhibition of osteoclast-mediated bone resorption, thereby preserving bone mass and reducing fracture risk [5]. However, despite their osteoprotective properties, bisphosphonates have also been associated with adverse effects such as medication-related osteonecrosis of the jaw (MRONJ), particularly following systemic administration and invasive dental procedures. Therefore, further investigation of their local biological effects in periapical tissues remains important [6]. Zoledronate, a potent nitrogen-containing bisphosphonate, exhibits strong antiresorptive effects through suppression of osteoclast activity and is widely used in clinical practice [7]. In addition to their direct effects on bone metabolism, bisphosphonates have been shown to modulate the local immune microenvironment by influencing cytokine expression and inflammatory cell recruitment. These combined osteoimmunomodulatory properties suggest that bisphosphonates may contribute to the regulation of inflammatory bone remodeling in periapical lesions [8]. Accordingly, local administration of bisphosphonates has been investigated as a potential approach for achieving targeted osteoprotective and immunomodulatory effects while reducing systemic exposure and associated adverse effects [9].

Nevertheless, the local effects of bisphosphonates on periapical lesion progression and bone metabolism remain insufficiently explored, particularly with regard to immune-regulatory mechanisms that may influence both their biological responses and potential adverse effects [10]. Investigating these effects in immunologically distinct rats could provide valuable insights into the osteoimmunomodulatory actions of locally applied bisphosphonates and their potential to modulate inflammatory bone remodeling. To address this, the present study employed two immunologically distinct rat strains: Albino Oxford (AO) rats, which exhibit a predominant T helper 2 (Th2)-type immune response, and Dark Agouti (DA) rats, which are characterized by a T helper 1 (Th1)-dominant profile [11,12,13]. The use of these strains offers a unique experimental background to explore how different immune profiles may influence the local effects of zoledronate on periapical lesion development, inflammatory mediator expression, and bone remodeling.

The aim of this study was to investigate the effects of local zoledronate treatment during periapical lesion development on inflammatory and bone remodeling responses in two immunologically distinct inbred rat strains. By comparing these two strains, we aimed to elucidate how differences in immune profile may influence the biological effects of local zoledronate treatment on periapical inflammation and bone remodeling.

## 2. Materials and Methods

This study was conducted on 44 male DA and AO rats (8 weeks old) obtained from the Military Medical Academy (Belgrade, Serbia) with body weights appropriate for their age and strain (DA: approximately 180–220 g; AO: approximately 200–250 g). All animals were clinically healthy and free from any signs of systemic disease prior to the experimental procedures. Animals were housed under standard laboratory conditions (25 °C, 50% humidity, and 12/12 h light/dark cycle) with free access to food and water. All experimental procedures were approved by the Ethics Committee of the Faculty of Medical Sciences, University of Kragujevac (approval no. 01-6885; 15 June 2021), and performed in accordance with EU Directive 2010/63/EU, Council Directive 86/609/EEC, the principles of Good Laboratory Practice, and the ARRIVE guidelines [14]. The study was designed according to the principles of the 3Rs (replacement, reduction, and refinement), with all efforts made to minimize animal suffering and reduce the number of animals used. Animals were monitored daily for general health status, bodyweight changes, signs of distress, and behavioral alterations throughout the experimental period. No severe adverse events requiring early euthanasia were observed during the study.

### 2.1. Induction of Periapical Lesions

Periapical lesions were induced by exposing the pulp of the right first mandibular molars under general anesthesia with ketamine (80 mg/kg) and xylazine (10 mg/kg), administered intraperitoneally. Pulp chambers were accessed using a high-speed dental handpiece with a sterile, round bur; then, canals were explored with a size 08 endodontic file. Pulp tissue was left exposed to the oral cavity [11].

### 2.2. Administration of Bisphosphonate

Animals were randomly assigned to one of four experimental groups (*n* = 11/group): DA rats receiving submucosal zoledronate (0.15 mg/kg), DA rats receiving submucosal saline (0.9% NaCl) as the control group for the DA strain, AO rats receiving submucosal zoledronate (0.15 mg/kg), and AO rats receiving saline administered in the same volume as the control group for the AO strain [15]. Randomization was performed using a simple random allocation method. Injections were administered locally into the periapical region on days 0, 7, 14, and 21 following pulp exposure during lesion development. The experimental period lasted 28 days [16]. Animals were monitored daily throughout the study period. On day 28, animals were anesthetized and euthanized by decapitation, and blood samples were collected from the cervical vessels. Mandibles were subsequently dissected for further analyses.

### 2.3. Sample Allocation

Radiographic analysis was performed using representative samples (*n* = 5 per group). Histological and immunohistochemical analyses were conducted on 5 specimens per group. For gene expression analysis (qRT-PCR), 6 animals per group were used. Blood samples were collected from all animals, and systemic oxidative stress parameters were analyzed in 11 animals per group. The distribution of animals across analyses was predefined to allow for separate tissue processing for molecular, histological, and biochemical assessments. The number of samples used for each analysis was determined according to the specific requirements of each methodological approach. Representative specimens of adequate quality were selected for radiographic, histological, immunohistochemical, and qRT-PCR analyses, and the inclusion of five or six animals per group was considered sufficient to address the objectives of these assessments while avoiding unnecessary consumption of tissue samples, in accordance with the principle of Reduction within the 3Rs framework. Group allocation was known to the investigator performing the experimental procedures, while radiographic, histological, and immunohistochemical outcome assessments were conducted by an investigator blinded to the experimental groups. Molecular and biochemical analyses were performed using coded samples to minimize bias during data acquisition and analysis.

No predefined exclusion criteria for animals or data points were established prior to the study. All animals that underwent the experimental procedures were included in the analyses. No animals, experimental units, or data points were excluded from the final analysis.

### 2.4. Radiographic Analysis

Hemimandibles from each rat strain were isolated and radiographically imaged using a Sirona Dental Systems device (Bensheim, Germany) at a standardized angle and fixed distance from the sensor. Radiographic settings were 70 kVp and 7 mA, with an exposure time of 0.12 s. Images were processed and analyzed using ImageJ software (version 1.53, National Institutes of Health, Bethesda, MD, USA). Calibration was performed using the known length of the endodontic instrument (21 mm) included in each radiograph. The periapical lesion area was manually delineated by a blinded examiner and quantified in square millimeters (mm^2^). Radiographic pixel intensity within the lesion area was additionally assessed as an indirect indicator of mineral density, with results reported as percentage values [17]. Following radiographic evaluation, tissue samples were processed for histopathological and immunohistochemical analyses.

### 2.5. Histological and Immunohistochemical Analysis

The previously radiographed hemimandibles were fixed in 4% paraformaldehyde in phosphate-buffered saline (PBS), decalcified in 3% formic acid, and embedded in paraffin. Longitudinal sections (5 μm thick) were prepared and stained with hematoxylin and eosin for histological evaluation [18]. Sections encompassing the distal root of the first mandibular molar and passing through or near the apical foramen were selected for histomorphometric analysis. Immunohistochemical analysis was performed on paraffin-embedded sections to evaluate osteocalcin expression in periapical tissues. Sections were incubated with a primary rabbit polyclonal antibody against osteocalcin (BGLAP) (Elabscience, Wuhan, China), followed by visualization using a horseradish peroxidase/3,3′-diaminobenzidine (HRP/DAB) detection kit (Abcam, Cambridge, UK) according to the manufacturer’s instructions. Negative controls were processed identically, with omission of the primary antibody. Tissue sections were imaged using a digital microscope (Olympus BX51, Tokyo, Japan), and the periapical lesion area (mm^2^), together with the osteocalcin-positive area fraction (%), was quantified using ImageJ software (version 1.53). Quantification was performed using standardized threshold settings applied uniformly to all analyzed sections [19]. All histological and immunohistochemical analyses were performed by a blinded examiner.

### 2.6. Gene Expression of Inflammation and Bone Markers by Quantitative Real-Time PCR

Quantitative real-time PCR (qRT-PCR) was used to determine the expression of cytokine- and bone-resorption-related genes in periapical lesions [10]. For this analysis, six animals per experimental group were used (total of 12 AO and 12 DA rats). Periapical lesions were carefully dissected from the right hemimandible and immediately immersed in RNAlater^®^ stabilization solution (Thermo Fisher Scientific, Waltham, MA, USA) to preserve RNA integrity, then stored at −80 °C until processing.

Total RNA was extracted from homogenized tissue using TRIzol™ reagent (Thermo Fisher Scientific, USA) according to the manufacturer’s instructions. The purity and concentration of RNA were assessed spectrophotometrically (Nano Drop, Thermo Fisher Scientific, USA), and only samples with an A260/A280 ratio between 1.8 and 2.0 were used for further analysis. One microgram of total RNA was reverse-transcribed into complementary DNA (cDNA) in a 20 µL reaction volume using a commercially available reverse transcription kit (RevertAid™ H Minus First Strand cDNA Synthesis Kit; Thermo Fisher Scientific, USA) [20].

qRT-PCR reactions were performed in a 20 µL total volume containing 0.1 µg of cDNA, SsoAdvanced Universal SYBR Green Supermix (Bio-Rad Laboratories, Hercules, CA, USA), and specific primers for the target genes (listed in Table 1).

Amplifications were carried out in a CFX96 thermal cycler (Bio-Rad Laboratories, USA) with the following cycling conditions: initial denaturation at 95 °C for 30 s, followed by 40 cycles of denaturation at 95 °C for 15 s and annealing and extension at 60 °C for 30 s. A melt-curve analysis was performed at the end of each run to confirm the specificity of the amplified products. Relative gene expression levels were calculated using the 2^−ΔΔCt^ method, normalizing the expression of target genes to β-actin as the housekeeping gene. All reactions were run in triplicate to ensure reproducibility, and negative controls (no-template controls) were included in each run to exclude contamination [21].

### 2.7. Oxidative Stress Markers in Rats with Periapical Lesions

Plasma samples were analyzed for biomarkers of oxidative stress, including lipid peroxidation (measured as TBARS) [22], nitric oxide (NO) production estimated via nitrite (NO_2_^−^) levels using a Griess reagent-based assay [23], superoxide anion radicals (O_2_^−^), and hydrogen peroxide (H_2_O_2_). In erythrocyte lysates, the activities of key antioxidant enzymes were measured, including catalase (CAT), superoxide dismutase (SOD), and reduced glutathione (GSH). All parameters were quantified spectrophotometrically (UV-1800, Shimadzu, Kyoto, Japan) at their respective wavelengths [24].

### 2.8. Statistical Analysis

Statistical analysis was performed using IBM SPSS Statistics software, version 26. The normality of the data distribution was assessed using the Kolmogorov–Smirnov and Shapiro–Wilk tests. Depending on whether the data followed a normal distribution, appropriate parametric or non-parametric tests were applied for group comparisons. Homogeneity of variances was evaluated using Levene’s test. For normally distributed data with homogeneous variances, one-way ANOVA was used for group comparisons, followed by Tukey’s HSD test for post hoc analysis. When the data were normally distributed but violated the assumption of homogeneity of variances, Welch’s ANOVA was applied, followed by the Games–Howell post hoc test. For data that did not follow a normal distribution, non-parametric tests were employed, including the Kruskal–Wallis test for comparisons among multiple groups and the Mann–Whitney U test for comparisons between two groups. Data are presented as mean values ± standard deviation. Statistical significance was defined as *p* < 0.05 [25,26].

Sample size was calculated using G*Power 3.1, assuming α = 0.05 and power = 0.80 [27]. Based on a previously published study with a similar experimental design [17], a minimum of seven animals per group was considered sufficient to detect statistically significant differences. To increase statistical robustness and ensure the sufficiency of biological material for multiple planned outcome measures, 11 animals were included in each group. Following euthanasia, animals were allocated to specific analyses according to tissue requirements and processing protocols. Radiographic, histological, and immunohistochemical analyses were performed in five animals per group; qRT-PCR analysis in six animals per group; and systemic redox analyses in all animals.

## 3. Results

### 3.1. Radiographic Assessment of Zoledronate Effects in Periapical Lesions of AO and DA Rats

**Figure 1:**
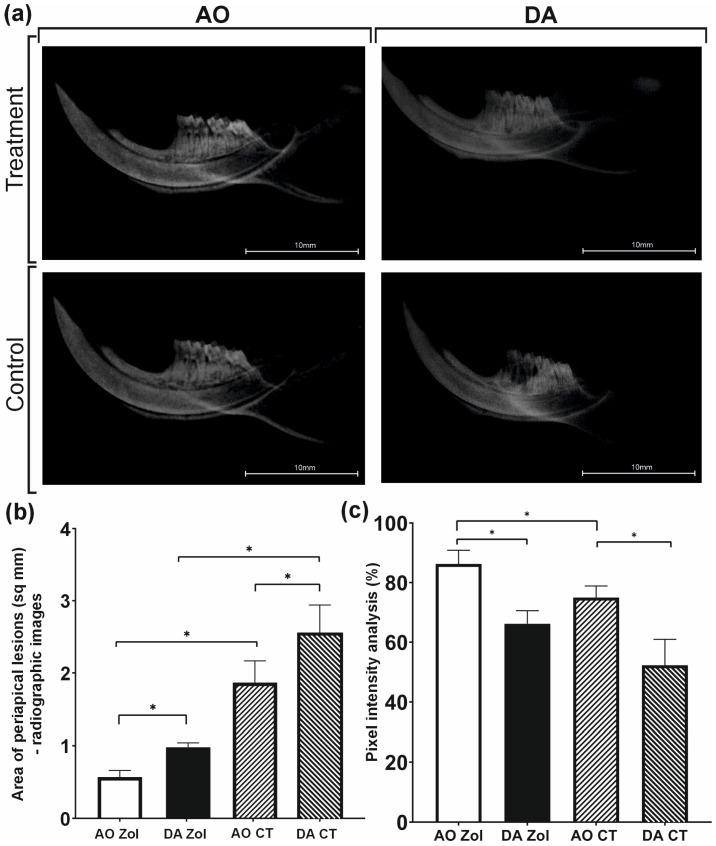
Radiographic analysis of periapical lesions in AO and DA rats. (**a**) Representative mandibular radiographs of zoledronate- and saline-treated AO and DA rats. (**b**) The periapical lesion area was significantly reduced following zoledronate treatment in both strains (*p* < 0.05), with smaller lesions in treated AO rats compared with treated DA rats (*p* < 0.05). (**c**) Pixel intensity analysis (%) showed significantly higher pixel-intensity values in zoledronate-treated AO rats compared with saline controls (*p* < 0.05), while no significant difference was observed between zoledronate- and saline-treated DA rats (*p* > 0.05). AO rats exhibited higher grayscale values than DA rats under both conditions (*p* < 0.05). * *p* < 0.05, statistically significant difference.

Radiographic analyses were performed to evaluate the effects of local zoledronate treatment on periapical lesion development. Radiographic assessment demonstrated smaller periapical lesion areas in zoledronate-treated animals compared with their respective controls in both AO and DA strains (both *p* = 0.008). Comparison between zoledronate-treated groups revealed significantly smaller lesions in AO rats than in DA rats (*p* = 0.008), suggesting differences in lesion progression between the strains. Radiographic grayscale analysis was additionally performed to assess changes in mineral density within the periapical region. AO rats exhibited significantly higher grayscale density values than DA rats following zoledronate treatment (*p* < 0.001), suggesting reduced bone resorption in the AO strain. Detailed radiographic comparisons between experimental and control groups are presented in Figure 1.

### 3.2. Zoledronate Treatment Was Associated with Smaller Periapical Lesions and Higher Osteocalcin Expression in AO Rats

**Figure 2:**
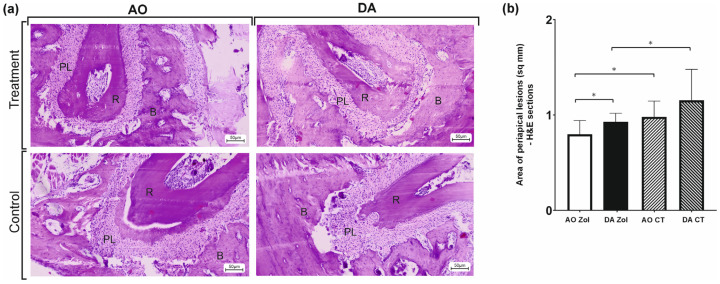
Histological evaluation of periapical lesions in AO and DA rats. (**a**) Representative hematoxylin–eosin-stained sections of periapical regions from saline- and zoledronate-treated Albino Oxford (AO) and Dark Agouti (DA) rats. Periapical lesions (PLs) are located around the root apex (R) and adjacent alveolar bone (B). Scale bar = 50 µm. (**b**) Histomorphometric analysis of the periapical lesion area (mm^2^) in H&E sections demonstrated significantly smaller lesions in zoledronate-treated animals compared with their respective saline-treated controls in both AO and DA strains (*p* < 0.05). In addition, zoledronate-treated AO rats exhibited significantly smaller lesions than zoledronate-treated DA rats (*p* < 0.05). * *p* < 0.05, statistically significant difference.

Consistent with the radiographic findings, histological examination demonstrated significantly smaller periapical lesions in zoledronate-treated AO rats compared with DA rats (*p* = 0.001), suggesting differences in histological responses between the strains. Additional intergroup comparisons are presented in Figure 2.

**Figure 3:**
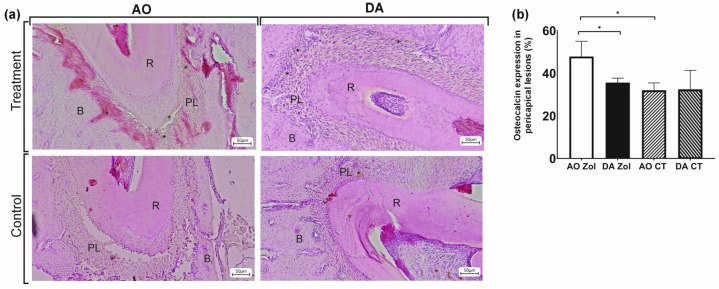
Immunohistochemical analysis of osteocalcin expression in periapical regions of AO and DA rats. (**a**) Representative sections from saline- and zoledronate-treated groups showing osteocalcin-positive staining predominantly in the alveolar bone (B) surrounding the root apex (R) and adjacent to the periapical lesion (PL). Scale bar = 50 µm. (**b**) Quantification of osteocalcin expression (%). Zoledronate treatment increased osteocalcin expression in AO rats compared with saline-treated controls (*p* < 0.05). In the DA strain, no significant difference was observed between zoledronate-treated and saline-treated animals (*p* > 0.05). A statistically significant difference was observed between the AO ZOL and DA ZOL groups (*p* < 0.05). * *p* < 0.05, statistically significant difference.

To further evaluate osteogenic activity within periapical lesions, osteocalcin expression was assessed by immunohistochemical analysis. Comparison of the zoledronate-treated groups revealed significantly higher osteocalcin expression in AO rats than in DA rats (*p* = 0.008), indicating increased osteogenic activity in the AO strain. Detailed comparisons between experimental and control groups are presented in Figure 3.

### 3.3. Effect of Zoledronate Treatment on the Expression of Th1-, Th2-, and Th17-Related Genes in Periapical Lesions of DA and AO Rats

Gene expression analysis was performed to assess cytokines associated with Th1, Th2, and Th17 immune responses and to characterize the immunomodulatory effects of zoledronate treatment within periapical lesions.

Comparison between zoledronate-treated groups revealed significantly higher expression of pro-inflammatory cytokines TNF-α and IL-1β in DA rats compared with AO rats (*p* < 0.001), whereas IFN-γ expression did not differ significantly between the strains (*p* = 0.999).

In contrast, analysis of immunosuppressive cytokine IL-10 revealed significantly higher expression in AO rats compared with DA rats following zoledronate treatment (*p* = 0.001), suggesting differences in regulatory immune responses between the strains. A similar pattern was observed for IL-4, whose expression was also significantly higher in AO rats than in DA rats (*p* = 0.002).

Regarding the Th17-associated cytokine, IL-17 expression tended to be lower in AO rats than in DA rats, although this difference did not reach statistical significance (*p* = 0.913).

**Figure 4:**
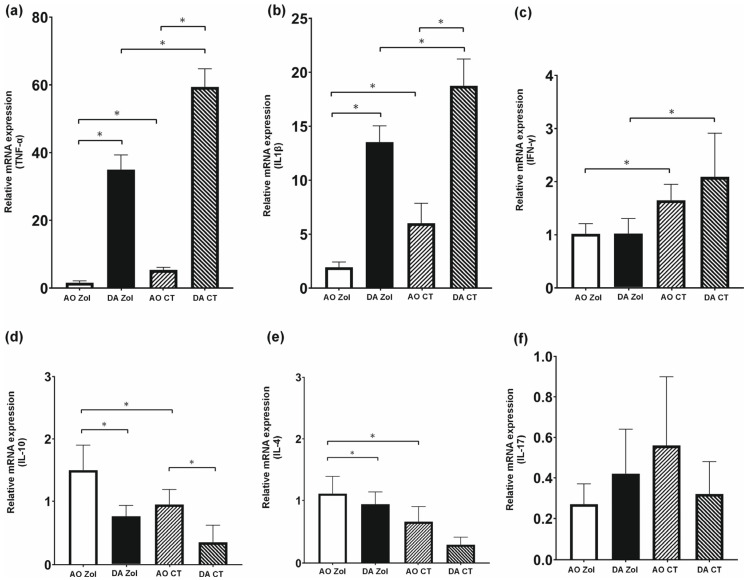
Relative mRNA expression of cytokines in periapical lesions of Albino Oxford (AO) and Dark Agouti (DA) rats following saline or zoledronate treatment. (**a**–**c**) Pro-inflammatory cytokines TNF-α, IL-1β, and IFN-γ. TNF-α and IL-1β expression differed significantly in all corresponding comparisons, including zoledronate-treated and saline-treated groups within each strain, as well as between AO and DA rats under both treatment conditions (*p* < 0.05). IFN-γ expression was significantly reduced in zoledronate-treated animals compared with saline controls in both AO and DA strains (*p* < 0.05). (**d**–**f**) Cytokine expression of IL-10, IL-4, and IL-17. IL-10 expression was significantly higher in zoledronate-treated AO rats compared with saline-treated rats (*p* < 0.05) and differed significantly between AO and DA rats under both treatment conditions (*p* < 0.05), while no significant difference was observed between zoledronate- and saline-treated DA rats (*p* > 0.05). IL-4 expression was significantly increased in zoledronate-treated AO rats compared with saline-treated rats (*p* < 0.05) and was higher in AO rats than in DA rats under zoledronate treatment (*p* < 0.05). IL-17 expression did not differ significantly between treatment groups or between strains (*p* > 0.05). Data are presented as mean ± SD. *p* < 0.05. * *p* < 0.05, statistically significant difference.

Detailed comparisons between experimental and control groups, as well as within strain analyses, are presented in Figure 4.

### 3.4. Bone Remodeling-Related Gene Expression in Periapical Lesions of AO and DA Rats

To further evaluate bone remodeling activity within periapical lesions and complement the observed changes in inflammatory cytokine expression, the expression of key regulators of osteoclastogenesis, RANKL and OPG, was assessed.

Comparison between zoledronate-treated groups revealed significantly lower expression of osteoclastogenic factor RANKL in AO rats compared with DA rats (*p* = 0.048). In contrast, the expression of anti-resorptive factor OPG tended to be higher in zoledronate-treated AO rats than in DA rats, although this difference did not reach statistical significance (*p* = 0.794).

**Figure 5:**
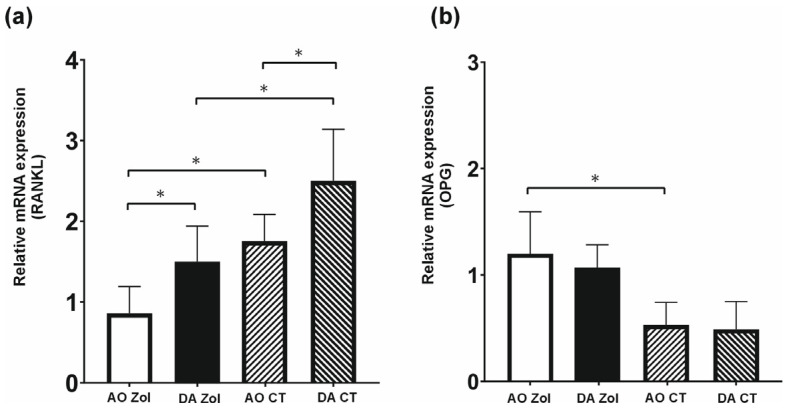
Effects of zoledronate treatment on bone remodeling markers in periapical lesions of AO and DA rats. (**a**) Relative mRNA expression of RANKL and (**b**) relative mRNA expression of OPG in zoledronate-treated (Zol) and control (CT) animals of both strains. RANKL expression differed significantly across all comparisons, including treatment effects within each strain and interstrain differences under both conditions (*p* < 0.05). OPG expression was significantly increased in zoledronate-treated AO rats compared with AO controls (*p* < 0.05), while no significant differences were observed in the DA strain or between AO and DA rats under either treatment condition (*p* > 0.05). Data are presented as mean ± SD. *p* < 0.05. * *p* < 0.05, statistically significant difference.

Detailed comparisons between experimental and control groups, as well as within-strain analyses, are presented in Figure 5.

### 3.5. Systemic Redox Status in AO and DA Rats Following Zoledronate Treatment

Systemic redox status was evaluated by analyzing selected pro-oxidative and antioxidant parameters in relation to the observed inflammatory and bone remodeling responses.

Analysis of pro-oxidative markers revealed significantly lower NO_2_^−^ levels in zoledronate-treated AO rats compared with DA rats (*p* < 0.001). In contrast, no statistically significant difference in H_2_O_2_ levels was observed between the strains (*p* = 0.994). Systemic O_2_^−^ levels were significantly higher in DA rats than in AO rats following zoledronate treatment (*p* = 0.022). No statistically significant differences were detected in TBARS levels between the strains (*p* = 0.482).

**Figure 6:**
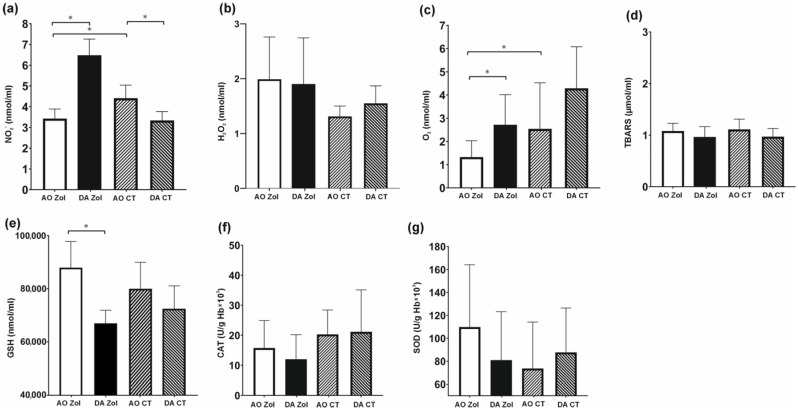
Systemic oxidative stress parameters in AO and DA rats following zoledronate treatment. Pro-oxidative markers (NO_2_^−^ (**a**), H_2_O_2_ (**b**), O_2_^−^ (**c**), and TBARS (**d**)) are presented in the upper row, and antioxidant defense parameters (GSH (**e**), CAT (**f**), and SOD (**g**)) are presented in the lower row. Zoledronate treatment produced a strain-dependent redox response. Nitrite (NO_2_^−^) levels significantly decreased in zoledronate-treated AO rats compared with saline controls, whereas zoledronate treatment in DA rats induced a significant increase. Furthermore, saline-treated AO rats exhibited significantly higher NO_2_^−^ concentrations compared with saline-treated DA rats, while zoledronate-treated DA animals showed significantly higher NO_2_^−^ levels than treated AO rats (*p* < 0.05). Hydrogen peroxide (H_2_O_2_) and TBARS showed no significant treatment-related or interstrain differences. Systemic O_2_^−^ levels were numerically reduced after zoledronate treatment in both strains without reaching statistical significance, although treated DA rats displayed higher values than treated AO rats (*p* < 0.05). Among antioxidant parameters, reduced glutathione (GSH) was significantly higher in zoledronate-treated AO rats compared with treated DA rats (*p* < 0.05), while catalase (CAT) and superoxide dismutase (SOD) activities did not differ significantly between treatment and strain groups. Data are presented as mean ± SD. *p* < 0.05. * *p* < 0.05, statistically significant difference.

Evaluation of antioxidant parameters demonstrated significantly higher GSH levels in zoledronate-treated AO rats compared with DA rats (*p* < 0.001). However, no statistically significant differences were observed in SOD (*p* = 0.399) or CAT (*p* = 0.808) levels between the strains. Detailed comparisons between experimental and control groups, as well as within-strain analyses, are presented in Figure 6.

## 4. Discussion

To the best of our knowledge, this is the first study to compare inflammatory responses and the effects of local zoledronate treatment during experimentally induced periapical lesion development in two immunologically distinct inbred rat strains. The present findings demonstrate differences between AO and DA rats in terms of inflammatory, osteogenic, and bone remodeling-related responses following local zoledronate treatment. Furthermore, we observed differences in the biological response to local bisphosphonate exposure depending on the host’s immune phenotype. AO rats exhibited smaller periapical lesions, higher osteocalcin expression, and increased expression of anti-inflammatory cytokines, whereas DA rats showed higher expression of pro-inflammatory cytokines and osteoclastogenic markers.

These findings suggest that differences in immune profile may influence the biological responses to local bisphosphonate administration during periapical lesion development and underscore the potential importance of the host immune background in shaping local osteoimmunological responses to bisphosphonate exposure.

Radiographic and histological analyses demonstrated smaller periapical lesions and reduced bone resorption in AO rats following local zoledronate treatment compared with DA rats, suggesting differences in biological responses between the strains. Although DA rats also exhibited smaller lesions following zoledronate treatment, these changes appeared to be less pronounced than those observed in AO rats. Previous studies have suggested that AO rats may exhibit lower susceptibility to periapical lesion development than DA rats [11], which could partially contribute to the smaller lesions observed in this strain. Still, local zoledronate treatment was associated with more favorable radiographic and histological findings in AO rats, supporting the potential influence of the immune background on osteoimmunological responses during periapical lesion development. These observations are consistent with previous experimental studies in rodent models [28,29,30]. For example, França et al. [15] reported smaller periapical lesions and reduced inflammatory changes following zoledronate treatment in Wistar rats based on radiographic and histological analyses. Similarly, Wayama et al. [28] and Silva et al. [29] demonstrated attenuation of periapical lesion progression following zoledronate exposure in ovariectomized rats, as confirmed by histological and micro-CT evaluation. In contrast to these studies, the present study used two-dimensional radiographic analysis rather than micro-CT evaluation, which represents a limitation of the study. Nevertheless, radiographic findings were supported by histological analyses, providing complementary assessment of periapical lesion changes. In addition, previous studies evaluating zoledronate treatment did not report osteonecrosis in roots, periodontal ligaments, or alveolar bone tissues [15,30]. However, further investigations are required to clarify the long-term biological effects and translational relevance of local bisphosphonate administration in endodontic inflammatory conditions. In particular, the potential relationship between local bisphosphonate exposure and MRONJ-related biological mechanisms should be further investigated, especially under conditions that more closely resemble clinical scenarios associated with impaired bone healing and chronic infection [30]. Considering the unique immunological and remodeling characteristics of jaw bones, the interaction between local inflammatory responses and bisphosphonate exposure may represent an important factor influencing susceptibility to osteonecrosis.

Immunohistochemical analysis further supported the morphometric and radiographic findings by demonstrating higher osteocalcin expression in zoledronate-treated AO rats compared with DA rats. In AO rats, local zoledronate treatment was associated with significantly increased expression of osteocalcin, a marker commonly associated with osteoblastic activity and bone formation [31]. In contrast, the DA strain did not exhibit a statistically significant increase in osteocalcin expression, although elevated staining was observed in some treated animals. Since osteocalcin expression may vary during different phases of bone remodeling, tissue harvesting on day 28 could have influenced the observed staining patterns. This time point was selected to ensure complete development of periapical lesions; however, earlier differences in osteogenic responses between the strains may have been less detectable at later stages of lesion progression [32]. No significant differences in osteocalcin expression were observed between AO and DA rats under baseline conditions, suggesting that the observed variations following zoledronate treatment were not solely attributable to baseline osteoblastic activity. The increased osteocalcin expression observed in AO rats may reflect differences in local osteogenic responses associated with the Th2-dominant immune profile of this strain, which has previously been associated with modulation of inflammatory responses and bone remodeling [11]. Collectively, these findings support the possibility that the host immune background may influence osteogenic responses during periapical lesion development under local bisphosphonate exposure [33]. The less pronounced osteocalcin response observed in DA rats may indicate differences in inflammatory and bone remodeling dynamics under Th1-dominant conditions [34,35].

Our findings suggest that local zoledronate treatment may influence inflammatory responses within periapical lesions by altering the expression of key pro- and anti-inflammatory cytokines, consistent with previous studies [36]. In AO rats, zoledronate treatment was associated with lower expression of pro-inflammatory mediators TNF-α and IL-1β, which coincided with smaller periapical lesions and reduced bone resorption compared with zoledronate-treated DA rats. The reduced expression of TNF-α and IL-1β observed in AO rats may reflect differences in local inflammatory regulation associated with the Th2-dominant immune profile of this strain [11]. TNF-α has been reported to synergize with IFN-γ under highly inflammatory conditions, contributing to osteoblast apoptosis and impaired bone formation [37,38]. In this context, IFN-γ expression was additionally evaluated. Although no statistically significant interstrain difference was observed between zoledronate-treated AO and DA rats, zoledronate treatment significantly reduced IFN-γ expression compared with saline-treated controls in both strains. These observations may indicate local modulation of inflammatory signaling within periapical tissues following zoledronate exposure. Supporting this interpretation, Kaur et al. [39] demonstrated that zoledronate reduced IFN-γ production in gingival tissues, suggesting a potential effect of bisphosphonates on local cytokine regulation.

Overall, these findings are in line with previous reports showing that zoledronate suppresses key inflammatory mediators involved in the progression of apical periodontitis, including marked reductions in IL-1β and TNF-α levels [36]. Through the downregulation of TNF-α, a cytokine known to stimulate osteoclast differentiation and activity [40], zoledronate effectively limits inflammatory bone resorption. Taken together, these results indicate that the anti-inflammatory and bone-preserving effects of zoledronate are closely linked to its ability to modulate local cytokine networks within periapical lesions. These findings suggest that zoledronate does not act solely through direct osteoclast inhibition. Based on the findings of the present study, together with evidence from previous reports, it may be assumed that zoledronate also indirectly modulates RANKL expression through its effects on inflammatory cytokines within the inflammatory microenvironment. However, further investigations of the underlying signaling pathways are needed to confirm this mechanism. Previous evidence suggests that nitrogen-containing bisphosphonates may influence inflammatory cytokine expression through modulation of osteoclast- and macrophage-associated inflammatory signaling [41].

In line with the obtained results, IL-17 expression remained largely unchanged following zoledronate treatment in both strains, and no significant interstrain differences were observed among zoledronate-treated animals. These findings suggest that IL-17-related responses may be less affected by zoledronate treatment under the presented experimental conditions and at the invested time point. IL-17 is predominantly produced by Th17 cells, whose activity may be less directly influenced by bisphosphonates compared with macrophage-associated inflammatory mediators such as TNF-α and IL-1β [42]. Accordingly, the absence of significant IL-17 modulation may indicate that zoledronate exerts more pronounced effects on other inflammatory cytokine pathways. Additionally, the selected experimental time point may have influenced the observed findings. It is possible that IL-17 expression was more prominent during earlier phases of lesion development and subsequently declined during lesion maturation [39]. Therefore, the lack of detectable IL-17 modulation in the present study does not exclude the potential involvement of Th17-associated responses in periapical inflammation.

With regard to IL-4 expression, interstrain comparison among zoledronate-treated animals demonstrated significantly higher IL-4 levels in AO rats compared with DA rats. Within-strain analyses further revealed significantly increased IL-4 expression in zoledronate-treated AO rats relative to saline-treated controls, whereas the increase observed in DA rats did not reach statistical significance. Importantly, no significant difference in baseline IL-4 expression was detected between saline-treated AO and DA groups, suggesting that the differences observed following zoledronate treatment were not solely attributable to inherent interstrain variation. Although IL-4 has been reported to exert context-dependent effects on osteoblast differentiation [43], it is also recognized for its role in the modulation of inflammatory bone resorption and macrophage-associated inflammatory responses. The increased IL-4 expression observed in AO rats following zoledronate treatment may reflect differences in anti-inflammatory responses associated with the Th2-dominant immune profile of this strain. This interpretation is supported by previous experimental studies demonstrating that IL-4 deficiency may exacerbate periapical lesion progression and inflammatory bone destruction [44]. Collectively, these findings suggest that IL-4-associated responses may contribute to inflammatory bone remodeling during periapical lesion development following local zoledronate treatment.

In contrast to Maia et al. [36], who did not observe significant changes in IL-10 expression following zoledronate treatment, the present study demonstrated increased IL-10 expression, particularly in AO rats. Interstrain comparison among zoledronate-treated animals revealed significantly higher IL-10 expression in AO rats compared with DA rats. Within-strain analysis further confirmed significant upregulation of IL-10 in AO rats relative to saline-treated controls, whereas zoledronate treatment induced only a modest and statistically non-significant increase in IL-10 expression in DA rats. Importantly, baseline IL-10 levels were also significantly higher in AO control rats compared with DA controls, suggesting differences in basal anti-inflammatory responses between the strains. The discrepancy between the present findings and those reported by Maia et al. [36] may be related to differences in experimental design, including the animal strain that was used, the inflammatory microenvironment, and the timing of tissue analysis. In contrast to conventional rat models, the present study employed immunologically distinct inbred strains that may exhibit different baseline cytokine profiles and immune responsiveness during periapical lesion development. IL-10 is a key regulatory cytokine involved in the suppression of pro-inflammatory mediator production and the modulation of osteoclast differentiation. Although IL-10 has been reported to exert context-dependent effects on osteoblastogenesis, its anti-inflammatory properties may contribute to the modulation of inflammatory bone remodeling within periapical lesions [45]. In the present study, the increased IL-10 expression observed in AO rats following zoledronate treatment may reflect differences in local inflammatory regulation associated with the Th2-dominant immune profile of this strain.

Together with the parallel increase in IL-4 expression discussed above, these findings suggest that local zoledronate treatment may be associated with enhanced anti-inflammatory responses in AO rats. In contrast, the less pronounced IL-10 response observed in DA rats may reflect differences in cytokine regulation associated with the host immune profile and could contribute to the less pronounced changes in lesion size observed in this strain.

To further evaluate the mechanisms associated with the bone-preserving effects of zoledronate in periapical lesions, key regulators of bone remodeling were analyzed. Interstrain comparison among zoledronate-treated animals demonstrated significantly lower RANKL expression in AO rats compared with DA rats. Within-strain analyses additionally showed significantly reduced RANKL expression in both AO and DA rats relative to their respective saline-treated controls, suggesting that suppression of osteoclastogenic signaling represents an important biological effect of zoledronate across different immune backgrounds. Comparison between saline-treated groups further revealed lower baseline RANKL expression in AO rats compared with DA rats, consistent with previous observations suggesting differences in inflammatory and osteoclastogenic responses between these strains. In contrast, OPG expression demonstrated a different pattern. Interstrain comparison among zoledronate-treated animals revealed slightly higher OPG expression in AO rats than in DA rats; however, this difference did not reach statistical significance. Within-strain analyses demonstrated significantly increased OPG expression in AO rats following zoledronate treatment compared with saline-treated controls, whereas changes observed in DA rats did not reach statistical significance. These findings are generally consistent with previous evidence suggesting that bisphosphonates exert antiresorptive effects, predominantly through the suppression of RANKL-associated osteoclastogenic signaling, while OPG-related changes appear to be less pronounced and potentially dependent on the local inflammatory and immune microenvironment [10,46]. Collectively, the present findings suggest that the biological effects of zoledronate in periapical lesions may involve the modulation of the RANKL/OPG regulatory axis, with suppression of RANKL expression representing the more prominent effect under the presented experimental conditions.

Zoledronate treatment was associated with strain-related differences in selected oxidative stress parameters. In AO rats, lower NO_2_^−^ levels suggested reduced systemic oxidative activity, whereas the opposite trend observed in DA rats may indicate that redox responses to bisphosphonate exposure are influenced by the host immune background [47]. In addition, interstrain comparison among zoledronate-treated animals revealed significantly lower systemic O_2_^−^ levels in AO rats compared with DA rats, further supporting differences in pro-oxidative status between the strains following treatment. These findings are consistent with previous evidence suggesting that bisphosphonates may exert context-dependent effects on oxidative and inflammatory pathways. At the same time, the relatively mild and predominantly non-significant changes observed in H_2_O_2_, TBARS, CAT, and SOD suggest that local zoledronate treatment was not associated with marked systemic oxidative imbalance under the presented experimental conditions. Interestingly, elevated GSH levels observed in AO rats following zoledronate treatment may reflect differences in antioxidant responses between the strains [11]. This interpretation is consistent with previous reports indicating that GSH levels may transiently increase under inflammatory or oxidative conditions before subsequent stabilization or decline over time [48,49,50]. Collectively, these findings suggest that oxidative responses associated with local zoledronate treatment may vary depending on the inflammatory and immune background during periapical lesion development.

Our findings further support the concept that the biological effects of local zoledronate treatment in periapical lesions may involve not only the modulation of osteoclast-associated bone resorption but also alterations in local inflammatory and osteoimmunological responses. Previous studies have primarily focused on the antiresorptive effects of bisphosphonates and the potential development of osteonecrosis, whereas the influence of the host immune profile on local biological responses has remained insufficiently explored [14,51,52,53].

Using immunologically distinct rat strains, this study identified differences in inflammatory, osteogenic, and bone remodeling responses following local zoledronate treatment. Compared with DA rats, AO rats exhibited reduced lesion progression, together with more favorable inflammatory and osteogenic characteristics. Overall, these findings suggest that the local effects of zoledronate during periapical lesion development may depend not only on antiresorptive activity but also on interactions between inflammatory signaling and bone remodeling pathways influenced by the host immune profile.

Although these findings provide novel insights into the immunomodulatory and osteogenic effects of locally administered zoledronate during periapical lesion development, several limitations should be acknowledged. First, the findings were obtained in an experimental rat model and, therefore, should be interpreted with caution when extrapolating the results to human periapical disease. Second, zoledronate was administered locally into the periapical region, whereas the drug is predominantly used systemically in clinical practice, which may limit the direct translational relevance of the findings. Third, the observation period was limited to 28 days and, thus, reflects only the early stages of periapical lesion development and bone remodeling. Furthermore, the present study evaluated the influence of zoledronate during lesion development rather than its therapeutic effect on already established lesions. Therefore, additional long-term studies are needed to assess the persistence of the observed effects, healing dynamics, and potential adverse outcomes associated with bisphosphonate use, including medication-related osteonecrosis of the jaw.

## 5. Conclusions

In conclusion, the present study highlights the potential importance of the host immune profile in determining the local effects of zoledronate treatment during periapical lesion development. Local zoledronate treatment was associated with the modulation of inflammatory, osteogenic, and bone remodeling pathways in both AO and DA rats. Although suppression of osteoclastogenic signaling was observed in both strains, differences in cytokine levels, osteocalcin expression, and oxidative stress parameters suggested variability in local biological responses depending on the immune profile. AO rats exhibited smaller periapical lesions, together with increased expression of anti-inflammatory cytokines and osteogenic markers, whereas DA rats demonstrated a comparatively less pronounced regenerative response.

Taken together, these findings support the concept that the immune background may influence local osteoimmunological responses to bisphosphonate exposure during periapical lesion development. Further experimental and translational studies are needed to clarify the underlying mechanisms and potential clinical relevance of local bisphosphonate administration in endodontic inflammatory conditions.

## Figures and Tables

**Table 1:** Forward and reverse primer sequences used for quantitative real-time PCR (qRT-PCR) analysis of inflammatory cytokines, RANKL, OPG, and β-actin.

Gene	Sense (Forward) Primer (5′→3′)	Antisense (Reverse) Primer (5′→3′)
*IL-4*	TGCACCGAGATGTTTGTACC	GGATGCTTTTTAGGCTTTCC
*IL-10*	CAGTCAGCCAGACCCACAT	GCTCCACTGCCTTGCTTT
*IL-17*	CTACCTCAACCGTTCCACT	TTCTCAGGCTCCCTCTTC
*TNF-α*	GTAGCCCACGTCGTAGCAAA	CCCTTCTCCAGCTGGAAGAC
*IFN-γ*	GCTAGATTCTGGTGACAGCTGGTG	CACCAGCTGTCACCAGAATCTAGC
*IL-1β*	TGATGTTCCCATTAGACAGC	GAGGTGCTGATGTACCAGTT
*β-actin*	ACGGTCAGGTCATCACTATCG	GGCATAGAGGTCTTTACGGATG
*RANKL*	CACAGCGCTTCTCAGGAGTT	GATGGTGAGGTGAGCAAACG
*OPG*	ACAGTTTGCCTGGGACCAAA	TCACAGAGGTCAATGTCTTGGA

## Data Availability

The data that support the findings of this study are available from the corresponding author upon reasonable request.
